# *Notes from the Field*: Varicella Fatality on a Cargo Vessel — Puerto Rico, 2015

**DOI:** 10.15585/mmwr.mm6615a4

**Published:** 2017-04-21

**Authors:** Misty Ellis, Carolina Luna-Pinto, Thomas George, Joanna J. Regan, Mona Marin, Adriana Lopez, Brenda Rivera-Garcia, Kara Tardivel

**Affiliations:** ^1^Division of Global Migration and Quarantine, National Center for Emerging and Zoonotic Diseases, CDC; ^2^Division of Viral Diseases, National Center for Immunization and Respiratory Diseases, CDC; ^3^Puerto Rico Department of Health, Epidemiology.

The U.S. Code of Federal Regulations (42 §71.21) requires that the master of a ship destined to a U.S. port of entry report certain illnesses, as well as any death onboard to the nearest CDC Quarantine Station (*1*). On December 30, 2015, the U.S. Coast Guard notified CDC of the death of a crew member of a foreign cargo vessel off the coast of Puerto Rico. Four days earlier, on December 26, the patient, a man aged 50 years from India, developed abdominal pain, headache, and fever (103.0°F [39.4°C]), followed by loose stools and pruritus. On December 28, a vesicular rash appeared on his face, neck, and shoulders. Medical consultants suspected varicella and recommended shipboard isolation. On December 29, the vesicles had begun to dry and scab, and he developed a nonproductive cough and reported chest congestion. On December 30, he had difficulty breathing and collapsed; cardiopulmonary resuscitation was unsuccessful. The Puerto Rico Department of Health was contacted to liaise with the medical examiner. Lung tissue and skin lesion specimens collected at autopsy were positive for varicella-zoster virus DNA by polymerase chain reaction at CDC. The cause of death was reported as varicella pneumonia. No other medical conditions were reported.

Per CDC recommendations, all 24 shipmates were considered contacts of the index patient; the master of the ship instituted daily temperature and rash surveillance for 21 days (i.e., one incubation period) after the death. On days 13 and 16 of surveillance, two crew members were sent home because of emergencies unrelated to varicella. San Juan and Houston CDC Quarantine Stations coordinated varicella vaccination for the 22 remaining and five new crew members boarding after the end of the 21-day surveillance, all of whom had unknown varicella immunity. Acyclovir was procured by the ship for treatment of possible additional cases; however, none occurred. 

Varicella, a highly contagious disease caused by the varicella-zoster virus, is transmitted by direct contact with vesicle fluid, or through breathing infectious droplets. Varicella is typically a mild disease; however, adults are at risk for more severe illness and have a higher incidence of complications, most commonly pneumonia (*2*). Adults who grew up in tropical countries or countries where varicella vaccination is uncommon might have increased varicella susceptibility (*3*). Varicella rarely results in death; mortality rates during 1990–1994 (before vaccine licensure) were 0.3 per 1,000,000 population among persons aged ≥20 years. Pneumonia was the most common cause of death in previously healthy persons with varicella in this age group (*4*). Before effective chemotherapy, a case fatality rate of 10%–30% was reported among adults with varicella pneumonia (*5*).

This investigation highlights the importance of early notification of illness or death to CDC by ships arriving to U.S. ports of entry, and the use of CDC’s varicella management guidance to prevent further transmission (*6*), including surveillance for febrile rash illness, isolation of cases, screening for varicella immunity, and vaccination of nonimmune persons. Collaboration with the U.S. Coast Guard was critical for expediting communication with the master of the ship. Assistance from the Puerto Rico Department of Health and the Puerto Rico Forensic Sciences Institute was instrumental in ensuring a thorough and timely investigation and public health response. Keeping a stock of acyclovir onboard was added to the CDC maritime varicella management guidance (*6*).
